# Supplementary material for: Induction of p53-Dependent Apoptosis by Prostaglandin A2
Source: Biomolecules. 2020 Mar 24;10(3):492. doi: 10.3390/biom10030492 (PMC7175137; doi:10.3390/biom10030492)
Supplement: Supplementary file 1 [file biomolecules-10-00492-s001.pdf]

Supplementary Figure Legends

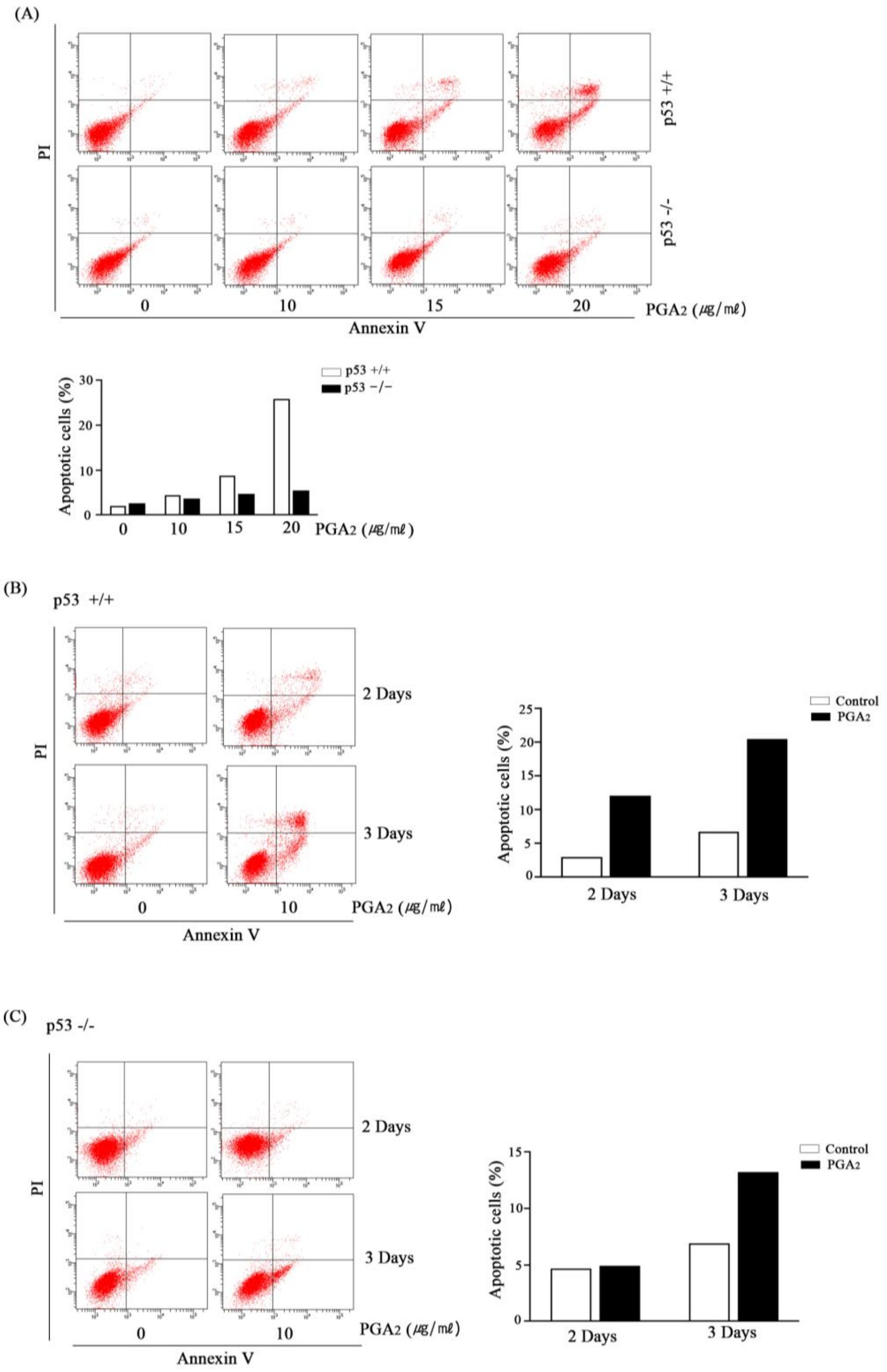

(D)

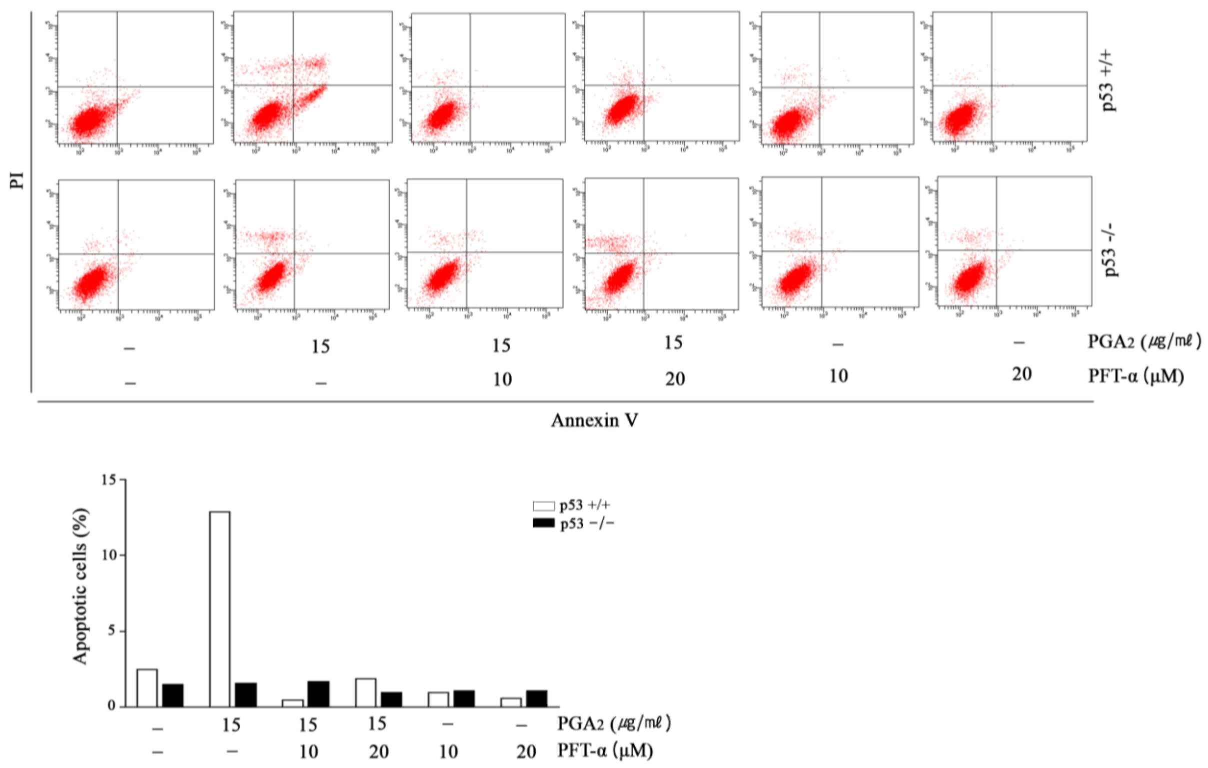

Figure S1. Induction of apoptosis by PGA_2 in HCT116 cells. (A) HCT116 and HCT116 p53-/- cells were treated with indicated concentrations of PGA_2 for 24 h. Cells were then harvested and subjected to annexin V assay. (B) HCT116 cells were treated with vehicle or PGA_2 (10 μg/ml) for 48 h and 72 h. Cells were then subjected to annexin V assay. (C) HCT116 p53-/- cells were treated with vehicle or PGA_2 (10 μg/ml) for 48 h and 72 h. Cells were then subjected to annexin-V assay. (D) HCT116 cells and HCT116 p53-/- cells treated with indicated concentrations of PFT-*α* for 1 h were incubated in the absence or presence of PGA_2 (15 μg/ml) for another 18 h. Cells were then subjected to annexin V assay.

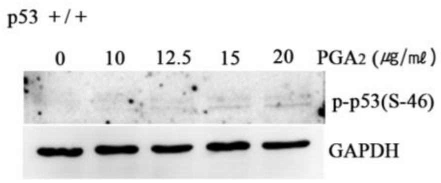

Figure S2. Phosphorylation of p53 at Ser-46 by PGA_2. HCT116 cells were treated with indicated concentrations of PGA_2 for 12 h and subjected to immunoblot analysis against phospho-p53 Ser-46 and GAPDH as an internal reference protein.

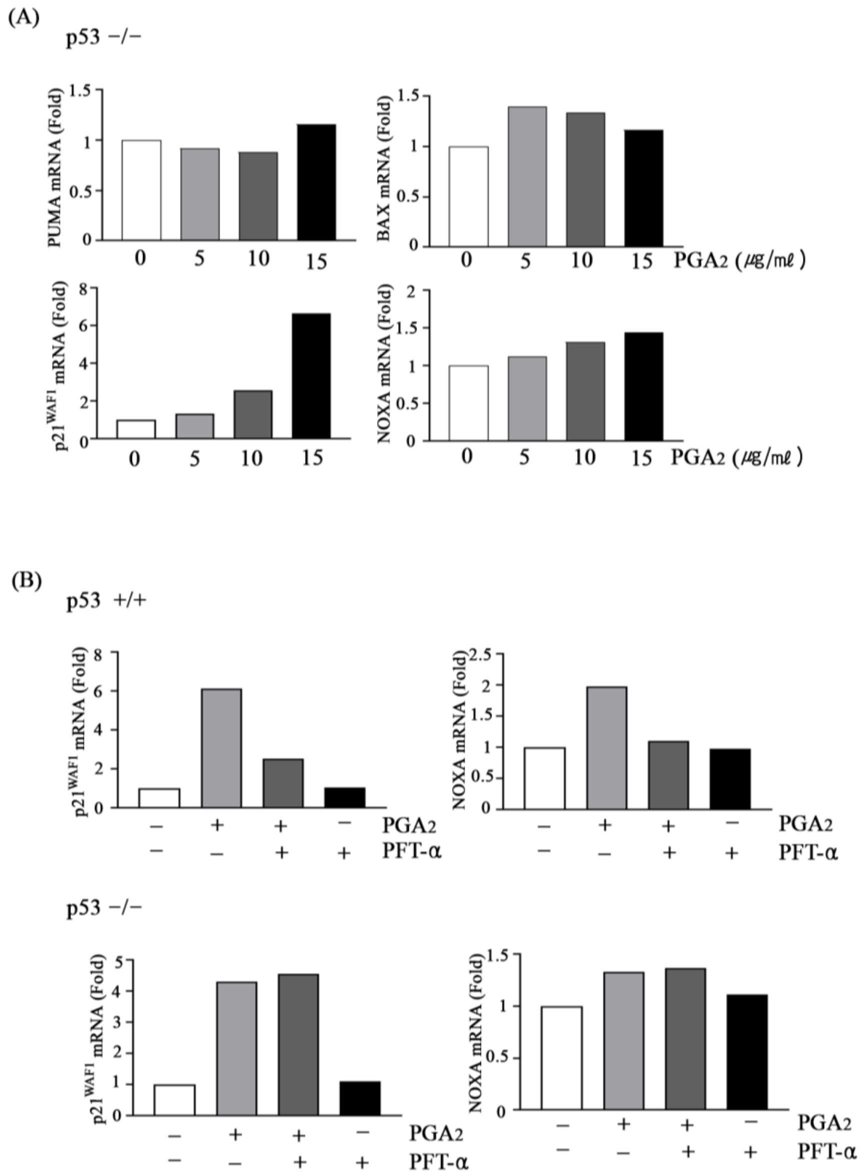

Figure S3. Measurement of p53 target genes. (A) Total cellular RNA of HCT116 p53-/- cells treated with indicated concentrations of PGA_2 for 12 h were subjected to qPCR against indicated genes using *GADPH* as an internal reference gene for normalization. (B) HCT116 and HCT116 p53-/- cells pretreated with vehicle or PFT-α for 1 h were incubated in the presence of vehicle or PGA2 (15 ug/ml) for another 12 h. Then, total cellular RNA were extracted and subjected to qPCR against $p21^{WAF1}$ and *NOXA* using *GAPDH* as an internal reference gene for normalization.

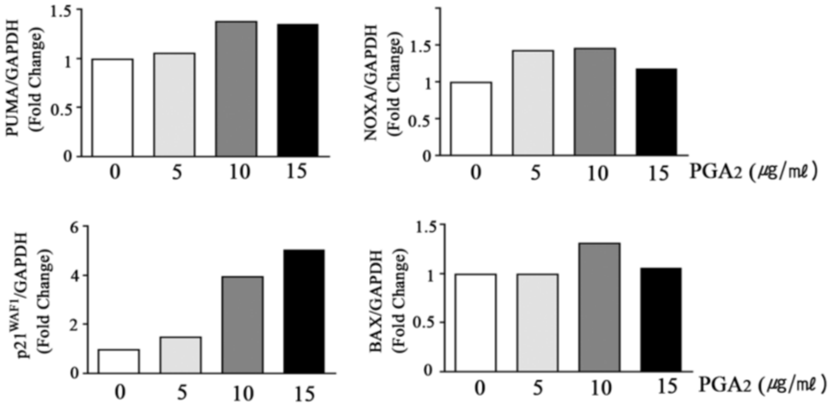

Figure S4. Densitometric analysis of p53 target genes. These graphs shows densitometric analysis of Figure 2B.

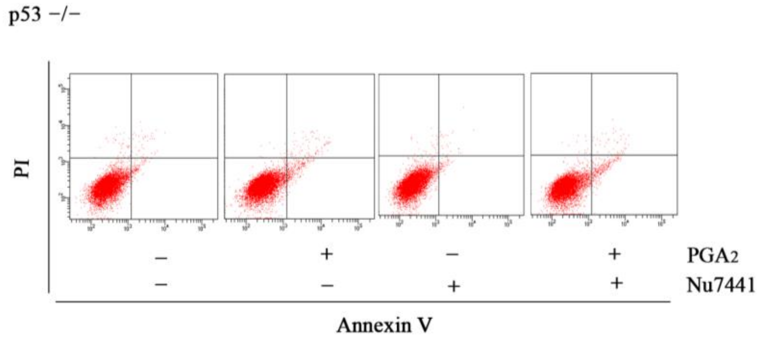

Figure S5. The effect of NU7441 on survival of HCT116 p53-/- cells. HCT116 p53-/- cells were treated with NU7441 for 1 h and then treated with vehicle or PGA$_2$ for another 18 h. Cells were then subjected to annexin V assay.

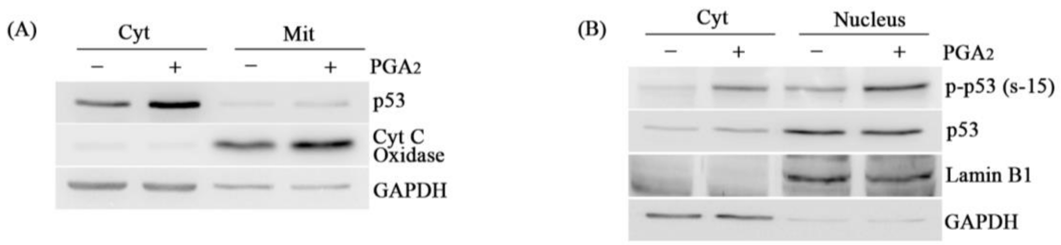

Figure S6. The subcellular localization of p53 expression in PGA$_2$-treated cells. (A) The cytosol (Cyt) and mitochondrial fractions (Mit) of HCT116 cells treated with vehicle or PGA$_2$ (15 μg/ml) for 18 h were subjected to immunoblot analysis against p53, cytochrome c oxidase (cyt c oxidase), and GAPDH. (B) The cytosol (Cyt) and nuclear fractions (Nucleus) of HCT116 cells treated with vehicle or PGA$_2$ (15 μg/ml) for 18 h were subjected to immunoblot analysis against phospho-p53 [Ser-15, p-p53 (s-15)], p53, lamin B1, and GAPDH. .

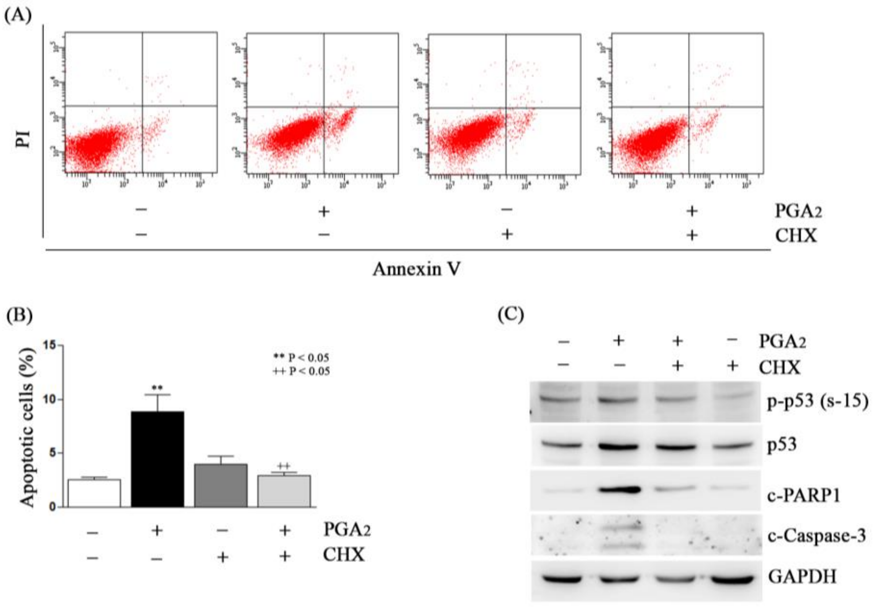

Figure S7. The effect of CHX on the PGA2-induced apoptosis in HCT116 cells. (A) HCT116 cells treated with CHX (1 μg/ml) for 1 h were incubated in the vehicle or PGA2 (15 μg/ml) for another 18 h. (A, B) Cells were then subjected to annexin V assay. The representative images (A) and statistical analysis (B) of three independent experiments were shown, respectively. The result of three independent annexin V assay was presented as mean ± SEM. (C) Whole cell lysates (WCLs) were prepared and subjected to immunoblot analysis against p53, phospho-p53 [Ser-15, p-p53 (s-15)], cleaved PARP1 (c-PARP1), cleaved caspase-3 (c-Caspase-3), and GAPDH used as an internal reference protein.

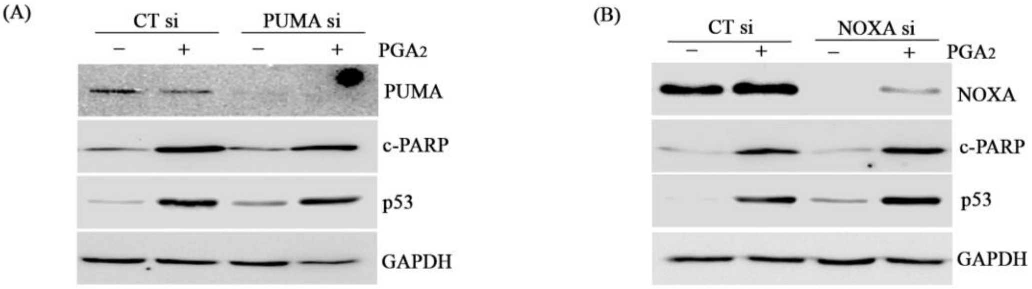

Figure S8. The effect of knockdown of PUMA and NOXA expression on PGA2-induced apoptosis. HCT116 cells transfected with siRNA against *PUMA* (A) or *NOXA* (B) for 24 h were incubated with vehicle or PGA2 for another 18 h. Cells were then subjected to immunoblot analysis against indicated proteins using GAPDH as an internal control.

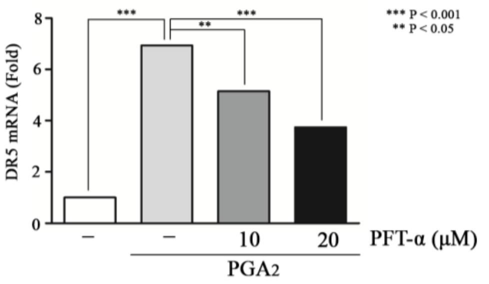

Figure S9. The effect of PFT-α on the PGA_2-induced increase of *DR5* mRNA. HCT116 cells pretreated with indicated concentrations of PFT-α for 1 h, were treated with vehicle or PGA_2 (15 μg/ml). At 18 h post-treatment of PGA_2, total cellular RNA was extracted and subjected to real-time qPCR analysis against *DR5* mRNA using *GAPDH* mRNA as an internal reference gene.

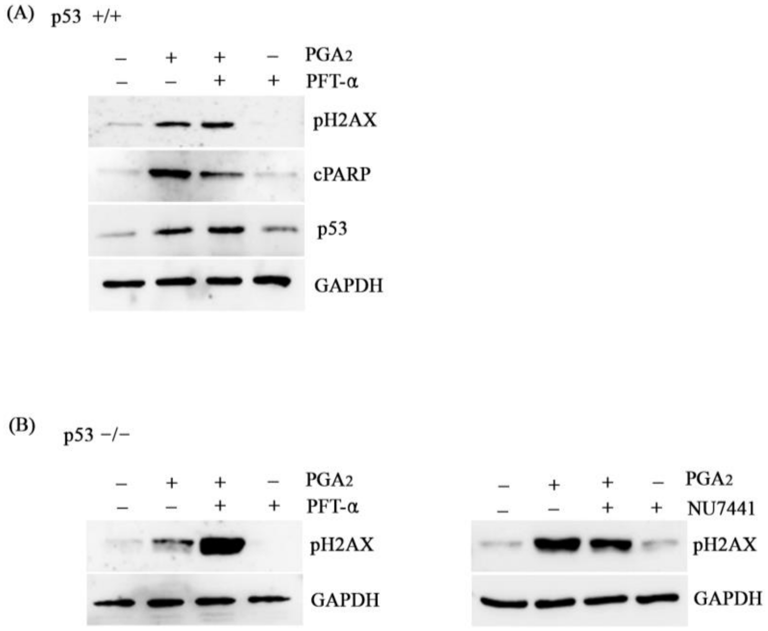

Figure S10. Phosphorylation of histone H2AX by PGA_2. (A) HCT116 cells pretreated with indicated concentrations of PFT-α for 1 h, were treated with vehicle or PGA_2 (15 μg/ml). At 18 h post-treatment of PGA_2, whole cell lysates were subjected to immunoblot analysis. (B) HCT116 p53-/- cells pretreated with PFT-α or NU7441 for 1 h, were treated with vehicle or PGA_2 (15 μg/ml). At 18 h post-treatment of PGA_2, whole cell lysates were subjected to immunoblot analysis against indicated proteins.

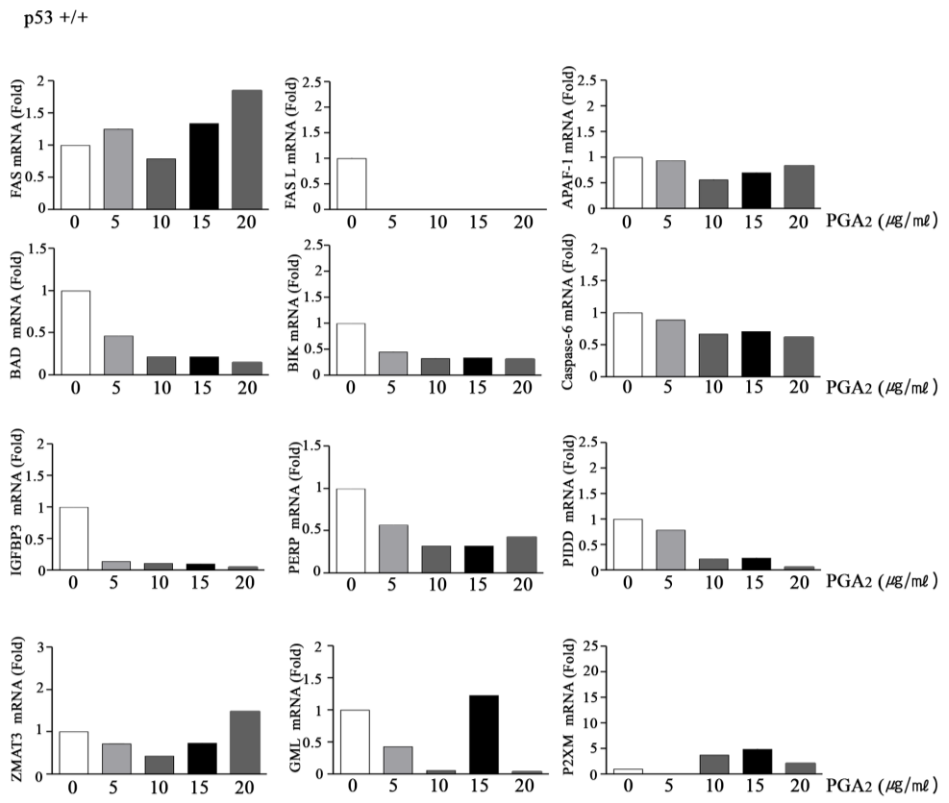

Figure S11. Measurement of various p53 target genes in PGA_2-treated cells. HCT116 cells were treated with indicated concentrations of PGA_2 for 12 h. Total cellular RNA were then prepared and subjected to qPCR against indicated genes using *GAPDH* as an internal reference gene. FAS-L, Fas ligand; IGFBP3, insulin-like growth factor-binding protein 3; GML, glycosylphosphatidylinositol- anchored molecule like; P2RX6, purinergic receptor P2X 6; PERP, p53 apoptosis effector related to PMP22; ZMAT3, zinc finger matrin-type 3; PIDD, p53-induced death domain protein 1; APAF1, apoptotic peptidase activating factor 1; BIK, BCL2 interacting killer; BAD, BCL2 associated agonist of cell death.

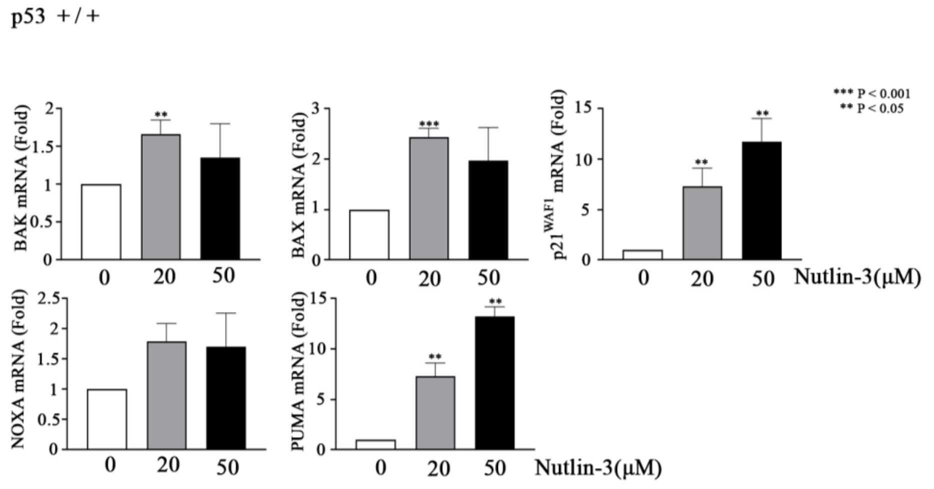

Figure S12. Induction of p53 target genes by nutlin-3. HCT116 cells were treated with indicated concentration of nutlin-3 for 18 h. Total cellular RNA were prepared and subjected to qPCR using *GAPDH* as an internal reference gene.

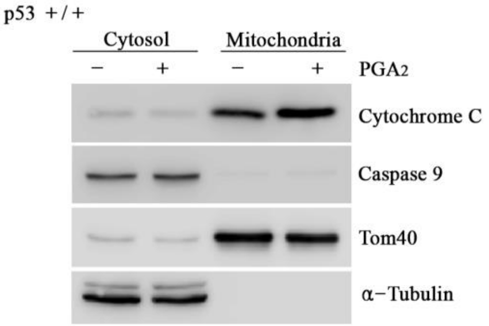

Figure S13. Analysis of cytochrome c release in PGA_2-treated cells. HCT116 cells treated with PGA_2 for 18 h were separated into cytosol and mitochondrial fractions. Each fraction was then subjected to immunoblot analysis against indicated proteins.

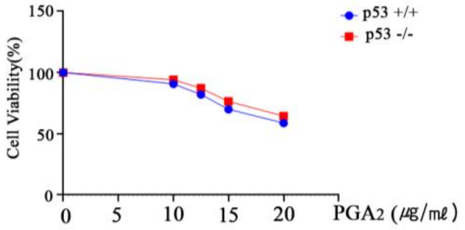

Figure S14. The effect of PGA_2 on the growth of HCT116 cells and HCT116 p53-/- cells. HCT116 cells and HCT116 p53-/- cells were treated with indicated concentrations of PGA_2 for 24 h. CCK-8 (cell counting kit-8) assay was performed to measure live cells.

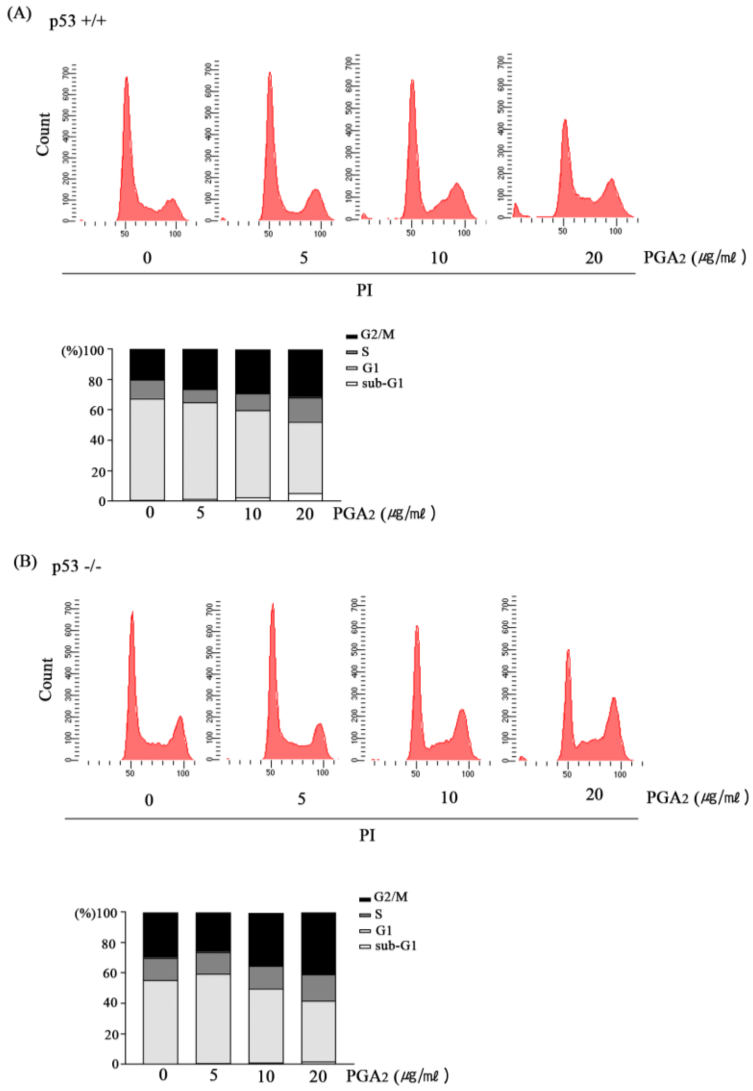

Figure S15. The effect of PGA_2 on the distribution of cell cycle of HCT116 cells and HCT116 p53-/- cells. HCT116 cells and HCT116 p53-/- cells were treated with indicated concentrations of PGA_2 for 24 h. Cells were then fixed and stained with propidium iodide. The cell cycle distribution was measured using FACS Canto II.
